# Isolation and Structural Determination of Two Novel Phlorotannins from the Brown Alga *Ecklonia kurome* Okamura, and Their Radical Scavenging Activities

**DOI:** 10.3390/md11010165

**Published:** 2013-01-18

**Authors:** Mari Yotsu-Yamashita, Sawako Kondo, Shinya Segawa, Yi-Chin Lin, Haruhiko Toyohara, Hisatomi Ito, Keiichi Konoki, Yuko Cho, Takafumi Uchida

**Affiliations:** 1 Graduate School of Agricultural Science, Tohoku University, Sendai 981-8555, Japan; E-Mails: sawakosn@yahoo.co.jp (S.K.); b1am1328@s.tohoku.ac.jp (S.S.); bellalin51244@gmail.com (Y.-C.L.), konoki@m.tohoku.ac.jp (K.K.); choyuko@biochem.tohoku.ac.jp (Y.C.); uchidat@biochem.tohoku.ac.jp (T.U.); 2 Graduate School of Agriculture, Kyoto University, Kyoto 606-8502, Japan; E-Mail: toyohara@kais.kyoto-u.ac.jp; 3 Beauty Care Products Division, Nagase & Co., Ltd., Kobe 651-2241, Japan; E-Mail: hisatomi.ito@nagase.co.jp

**Keywords:** phlorotannins, *Ecklonia kurome*, structural determination, NMR, radical scavenging activity

## Abstract

Two novel phlorotannins with a molecular weight of 974, temporarily named 974-A and 974-B, were isolated from the polyphenol powder prepared from the edible marine brown alga *Ecklonia kurome* Okamura, and their chemical structures were determined by spectroscopic method. The isolated yield of the total of 974-A and 974-B was approximately 4% (w/w) from the polyphenol powder. In 974-A, the carbon at the C2′ position in the A ring of phlorofucofuroeckol-A forms a C–C bond with the carbon at the C2″ position of the C ring of triphloretol-B, while in 974-B, phlorofucofuroeckol-B and triphloretol-B form a C–C bond in the same manner as in 974-A. These structures were supported by high resolution-MS/MS data. To evaluate the antioxidant activities, the 2,2-diphenyl-1-picrylhydrazyl (DPPH) radical scavenging assay and intracellular radical scavenging assay, using 2′,7′-dichlorofluorescin diacetate (DCFH-DA), were performed for 974-A, 974-B, and four known phlorotannins. The results of the DPPH assay showed that the IC_50_ values of 974-A, 974-B, phlorofucofuroeckol-A, and dieckol were significantly smaller than those of phlorofucofuroeckol-B, phloroglucinol, α-tocopherol, and ascorbic acid. Furthermore, the DCFH-DA assay suggested that 974-A, 974-B, and dieckol reduce intracellular reactive oxygen species most strongly among the tested compounds.

## 1. Introduction

**Figure 1:**
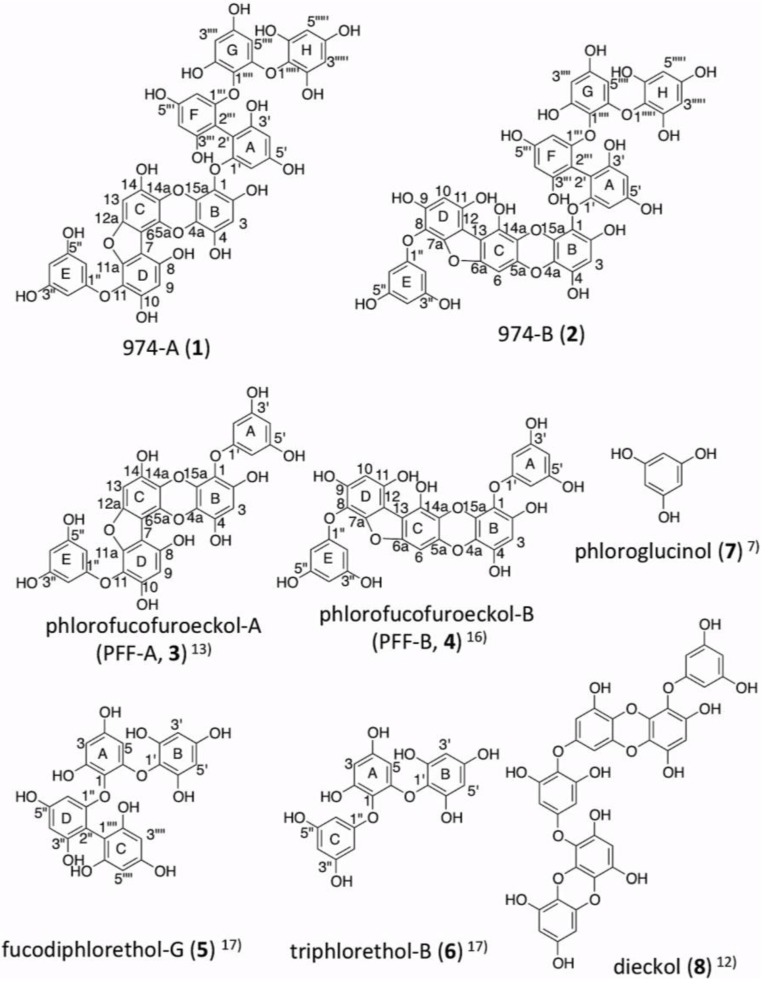
The structures of phlorotannins.**1** and **2** are the novel compounds reported inthis study, and the others are previously reported compounds.

Phlorotannins, the oligomers and polymers of phloroglucinol (1,3,5-trihydroxy benzene, Figure 1, **7**), widely occur among marine organisms, especially in brown algae. Based on the means of linkage, phlorotannins can be classified into four subclasses: phlorotannins with an ether linkage (fuhalols and phlorethols), with a phenyl linkage (fucols), with an ether and a phenyl linkage (fucophlorethols), and with a dibenzodioxin linkage (eckols and carmalols) [1]. Several remarkable bioactivities of phlorotannins have been reported, such as anti-inflammatory [2,3], antimicrobial [4], antiallergic [5], antioxidant [6], antitumor [7], angiogenesis [8], and tyrosinase inhibitory activity [9]. Recently, the crude polyphenol powder prepared from the edible marine brown alga *Ecklonia kurome* Okamura was reported to have inhibitory activities against α-amylase and α-glucosidase *in vitro*, and positive effects on oral carbohydrate tolerance test *in vivo* in genetically diabetic KK-A^y^ mice [10]. *E. kurome* is widely distributed on the south coast of Japan along the Pacific, and the midland coast of the Sea of Japan. This alga was previously reported to contain abundant phlorotannin derivatives with dibenzodioxin linkages, such as eckol, dieckol (Figure 1, **8**), 8,8′-bieckol and phlorofucofuroeckol-A (PFF-A, **3**) by Fukuyama *et al.* [11,12,13]. Regarding this background, we attempted to isolate and identify these major phlorotannins from the crude polyphenol powder from *E. kurome* for biological work. During the purification procedure, an unknown molecular ion ([M − H]^−^) at *m/z* 973 was detected using electrospray ionization mass spectrometry (ESI-MS) in high intensity. 

In this study, we isolated and determined the structures of two novel phlorotannins, temporarily named 974-A (**1**) and 974-B (**2**) of which molecular weights are 974 from the crude polyphenol powder prepared from *E. kurome*. Although two phlorotannins with the same molecular weight (974) have recently been reported from *Ecklonia cava* (2,7″-phloroglucinol-6,6′-bieckol [14] and pyrogallol-phloroglucinol-6,6′-bieckol [15]), the structures of these compounds are different from those we report in this study. Furthermore, the antioxidant activities of **1** and **2** were evaluated by measurement of 2,2-diphenyl-1-picrylhydrazyl (DPPH) radical scavenging activity, and by intracellular radical scavenging activity using 2′,7′-dichlorofluorescin diacetate (DCFH-DA), and compared with those of reported phlorotannins, phlorofucofuroeckol-A (PFF-A, **3**), phlorofucofuroeckol-B (PFF-B, **4**) [16] and dieckol (**8**) [12], purified from the same *E. kurome* polyphenol powder. We also examined the effects of **1** and **2** on the viability of three tumor cell lines. 

## 2. Results

### 2.1. Purification of 974-A (**1**) and 974-B (**2**), and Their Molecular Formulas

Air-dried *Ecklonia kurome* Okamura was extracted with ethanol/water (7:3, v/v), and the crude polyphenol powder was obtained from this extract by liquid chromatography on Diaion HP-20 (yield, 6.0%) [10]. The total polyphenol content of this crude polyphenol powder was previously reported to be over 70% [10]. From 500 mg of this crude polyphenol powder, 974-A (**1**) (6 mg) and 974-B (**2**) (9 mg) were purified by sequential chromatography on two reversed phase columns, Cosmosil 75C_18_-OPN and Mightysil RP-18 GP II. The isolated yield of the total of **1** and **2** was estimated as approximately 4% (w/w) from the polyphenols in the crude polyphenol powder. Three other previously reported phlorotannins, PFF-A (**3**) (4 mg) [13], PFF-B (**4**) (1 mg) [16] and dieckol (**8**) (5 mg) [12], were also isolated from the same source, and identified by comparison of their MS and NMR spectral data with reported ones. Details of the purification procedure are described in Experimental Section. 

The molecular formulas of **1** and **2** were determined to be C_48_H_30_O_23_ on the basis of their high-resolution (HR) ESI-TOF-MS spectra (**1**: [M − H]^−^ *m/z* 973.1069, calcd for C_48_H_29_O_23_ 973.1100, Δ −3.1 mmu, **2**: [M − H]^−^ *m/z* 973.1062, calcd for C_48_H_29_O_23_ 973.1100, Δ −3.8 mmu). (See Supplementary Material).

### 2.2. Determination of the Numbers of Hydroxyl Groups in 974-A (**1**) and 974-B (**2**)

First, the numbers of hydroxyl groups in **1** and **2** were determined by complete acethylation of these compounds with acetic anhydride and dehydrated pyridine (1:2, v/v). The molecular formulas of both of peracethylated **1** (**1a**) and **2** (**2a**) were determined to C_80_H_62_O_39_, indicating the presence of 16 hydroxyl groups in **1** and **2**(**1a**: HR-ESI-TOF-MS ([M + Na]^+^ *m/z* 1669.2770, calcd for C_80_H_62_NaO_39_1669.2760, Δ +1.0 mmu),** 2a**: HR-FAB-MS ([M + H]^+^ *m/z* 1647.2947, calcd for C_80_H_63_O_39_ 1647.2947, Δ +0.0 mmu)). Based on this data, **1** and **2** were distinguished from recently reported two phlorotannins with the same molecular formulas as those of **1** and **2**, 2,7″-phloroglucinol-6,6′-bieckol [14] and pyrogallol-phloroglucinol-6,6′-bieckol [15] from *Ecklonia cava*, because these compounds possess 14 and 15 hydroxyl groups, respectively. 

### 2.3. Determination of the Structure of 974-A (**1**)

^1^H NMR signals of **1** and **2** (especially **2**) were severely broadened in (CD_3_)_2_SO at 20–50 °C, while those in CD_3_OD appeared relatively sharp. Therefore, NMR spectra (^1^H, ^13^C, ^1^H–^1^H COSY, HSQC, HMBC) of **1** and **2** were mainly measured in CD_3_OD at 20 °C (See Supplementary Material). Although **3** and **4** are reported compounds, ^1^H and ^13^C NMR signals of **3** and **4** in CD_3_OD were also assigned in this study to compare with those of **1** and **2** (Table 1 and Table 2). In the ^1^H NMR spectra of **1**, three 1H singlet signals at δ 6.25, 6.40, 6.64, one 2H singlet signal at δ 5.93, three sets of *meta*-coupling doublet signals [δ 6.12 (d, *J* = 2.05 Hz)/δ 6.18 (d, *J* = 2.05), δ 6.20 (d, *J* = 2.35 Hz)/δ 5.90 (d, *J* = 2.35 Hz), δ 5.73 (d, *J* = 2.65 Hz)/δ 6.04 (d, *J* = 2.64 Hz)], and one set of *meta*-coupling 2H doublet signal and 1H triplet signal [δ 5.87 (d, *J* = 2.05)/δ 5.91 (t, *J* = 2.05)] (based on ^1^H–^1^H COSY correlations) were observed. The chemical shifts of the three singlet signals of **1** (δ 6.25, 6.40, 6.64) were close to those of **3** (δ 6.26, 6.40, 6.63) which were assigned to H3, H9 and H13 by comparison with previously reported NMR data of **3** in (CD_3_)_2_SO (δ 6.31, 6.44, 6.73) [13]. Furthermore, a 2H doublet signal at δ 5.87 (d, *J* = 2.05 Hz) coupling with a 1H triplet signal at δ 5.91 (t, *J* = 2.05 Hz) in **1**were also commonly shown in ^1^H NMR spectrum of **3** [δ 5.88 (d, *J* = 2.06 Hz), δ 5.94 (t, *J* = 2.06 Hz)], and these signals in **3** were assigned to H2′/H6′ and H4′ in the A ring, or H2″/H6″ and H4″ in the E ring (at this stage, A ring and E ring in **3** had an interchangeable assignment in CD_3_OD). Based on these data, **1**was presumed to contain the structural moiety of **3**. ^13^C NMR signals of **1** were assigned on basis of ^1^*J*_C,H_ and ^2,3,4^*J*_C,H_ correlations shown on their HSQC and HMBC spectra (*^n^J*_C,H_ 8 Hz) (HMBC correlations are listed in Table 1), respectively, and compared with ^13^C NMR signals of **3** (Table 1). The largest difference of ^13^C chemical shifts between **1** and **3** in A, B, C, D, E rings was only 0.8 ppm, except for the signals of C1, C2, and C3 in the A or E rings of **1**, supporting that **1** contains the structure of **3** (molecular formula: C_30_H_18_O_14_). Thus, the remaining part of **1** from **3** moiety was suggested to be composed of three aromatic rings (temporarily named as F, G, H rings) based on the molecular formula of **1**.

**Table 1:** NMR Spectroscopic Data^a^ for 974-A (**1**), PFF-A (**3**) and fucodiphlorethol G (**5**, part) in CD_3_OD.

	974-A (1)						PFF-A (3)		
ring	position	δ_C_	δ_H_ (mult, *J*)	HMBC ^b^			δ_C_	δ_H_ (mult, *J*)	Δδ_C_ (1−3)
A	1′	159.1					161.9		−2.8
	2′	102.4					95.4	5.96 (d, 2.06)	7.0
	3′	156.6					160.2		−3.6
	4′	98.4	6.18 (d, 2.05)	2′,3′,5′,6′			97.6	5.91 (t, 2.06)	0.8
	5′	159.6					160.2		−0.6
	6′	95.2	6.12 (d, 2.05)	1′,2′,4′,5′			95.4	5.96 (d, 2.06)	−0.2
B	1	124.2					124.7		−0.5
	2	147.8					148.3		−0.5
	3	99.3	6.25 (s)	1,2,4,4a,15a			99.3	6.26 (s)	0.0
	4	144.3					143.9		0.4
	4a	124.9					125.0		−0.1
	15a	138.2					138.4		−0.2
C	5a	135.2					135.3		−0.1
	6	105.2					105.3		−0.1
	12a	153.4					153.2		0.2
	13	96.1	6.64 (s)	5a,6,12a,14,14a			96.2	6.63 (s)	−0.1
	14	145.8					146.0		−0.2
	14a	127.6					128.1		−0.5
D	7	105.2					105.3		−0.1
	8	148.2					148.2		0.0
	9	99.9	6.40 (s)	7,8,10,11			99.9	6.40 (s)	0.0
	10	151.8					151.7		0.1
	11	122.3					122.3		0.0
	11a	151.2					151.2		0.0
E	1″	161.8					161.8		0.0
	2″,6″	95.3	5.87 (d, 2.05)	1″,3″,4″(H2″)			95.3	5.88 (d, 2.06)	0.0
	3″,5″	160.2					160.2		0.0
	4″	97.6	5.91 (t, 2.05)	2″,3″,5″,6″			97.7	5.94 (t, 2.06)	−0.1
					**fucodiphlorethol G (5, part) [17]**				
					**ring**	**position**	**δ_C_**	**δ_H_ (mult, *J*)**	**Δδ_C_ (1–5)**
F	1′′′	159.7			D	1″	159.4		0.3
	2′′′	102.6				2″	102.0		0.6
	3′′′	156.6				3″	159.2		−2.6
	4′′′	98.7	6.20 (d, 2.35)	2′′′,3′′′,5′′′,6′′′		4″	97.5	6.10 (d, 2.2)	1.2
	5′′′	159.7				5″	159.5		0.2
	6′′′	94.2	5.90 (d, 2.35)	1′′′,2′′′,4′′′,5′′′		6″	94.3	6.03 (d, 2.2)	−0.1
G	1″″	124.9			A	1	124.9		0.0
	2″″	151.9				2	152.0		−0.1
	3″″	97.7	6.04 (d, 2.64)	1″″,2″″,4″″,5″″		3	98.0	6.02 ^c^ (d, 2.7)	−0.3
	4″″	156.6				4	157.5		−0.9
	5″″	94.4	5.73 (d, 2.65)	1″″,3″″,4″″,6″″		5	94.5	5.69 ^c^ (d, 2.7)	0.1
	6″″	153.9				6	153.7		0.2
H	1′′′′′	124.2			B	1′	124.3		−0.1
	2′′′′′,6′′′′′	152.3				2′,6′	152.1		0.2
	3′′′′′,5′′′′′	96.4	5.93 (s)	1′′′′′,2′′′′′,4′′′′′(H3′′′′′)		3′,5′	96.0	5.91 (s)	0.4
	4′′′′′	156.5				4′	157.9		−1.4

^a^ ^1^H NMR (600 MHz), ^13^C NMR (151 MHz), chemical shift (ppm), *J* (Hz), the signals of CHD_2_OD (δ_H_ 3.30) and ^13^CD_3_OD (δ_C_ 49.0) were used as internal references; ^b^Correlations are from proton to carbon; ^c^ In ref. [17], the signals were oppositely assigned.

**Table 2:** NMR Spectroscopic Data^a^ for 974-B (**2**), PFF-B (**4**) and fucodiphlorethol G (**5**, part) in CD_3_OD.

	974-B (2)						PFF-B (4)		
ring	position	δ_C_	δ_H_ (mult, *J*)	HMBC ^b^			δ_C_	δ_H_ (mult, *J*)	Δδ_C_ (2–4)
A	1′	159.0					161.8		−2.9
	2′	102.5					95.1	5.97 (d,205)	7.3
	3′	156.9					159.9		−3.1
	4′	98.4	6.20 (d,2.05)	2′,3′,5′,6′			97.2	5.92 (t,2.05)	1.1
	5′	159.6					159.9		−0.4
	6′	95.2	6.15 (d,2.05)	1′,2′,4′,5′			95.1	5.97 (d 2.05)	0.0
B	1	124.0					124.3		−0.4
	2	147.0					147.5		−0.6
	3	99.6	6.17 (s)	1,2,4,4a,15a			99.2	6.16 (s)	0.3
	4	143.9					143.5		0.3
	4a	125.3					125.2		0.0
	15a	138.0					ND ^c^		ND ^c^
C	5a	142.9					143.0		−0.2
	6	92.7	6.69 (s)	5a,6a,13,14,14a			92.0	6.66 (s)	0.6
	6a	152.7					152.3		0.3
	13	109.9					109.7		0.1
	14	138.3					138.2		0.0
	14a	127.4					127.6		−0.3
D	7a	150.9					150.7		0.1
	8	122.1					122.2		−0.2
	9	151.6					151.3		0.2
	10	99.5	6.38 (s)	7a,8,9,11,12,13			98.9	6.37 (s)	0.5
	11	148.4					147.6		0.7
	12	106.4					106.7		−0.4
E	1″	161.9					161.7		0.1
	2″,6″	95.3	5.87 (d,2.06)	1″,3″,4″(H2″)			94.9	5.87 (d,2.03)	0.3
	3″,5″	160.2					160.0		0.1
	4″	97.6	5.91 (t,2.05)	2″,3″,5″,6″			97.2	5.94 (t,2.05)	0.3
					**fucodiphlorethol G (5, part) [17]**				
					**ring**	**position**	**δ_C_**	**δ_H_ (mult, *J*)**	**Δδ_C_ (2–5)**
F	1′′′	159.2			D	1″	159.4		−0.2
	2′′′	102.7				2″	102.0		0.7
	3′′′	157.5				3″	159.2		−1.7
	4′′′	98.6	6.22 (d, 2.06)	2′′′,3′′′,5′′′,6′′′		4″	97.5	6.10 (d, 2.2)	1.1
	5′′′	159.7				5″	159.5		0.2
	6′′′	94.2	5.89 (d, 2.05)	1′′′,2′′′,4′′′,5′′′		6″	94.3	6.03 (d, 2.2)	−0.1
G	1″″	124.7			A	1	124.9		−0.2
	2″″	152.2				2	152.0		0.2
	3″″	97.6	6.05 (d, 2.64)	1″″,2″″,4″″,5″″		3	98.0	6.02 ^d^ (d, 2.7)	−0.4
	4″″	156.6				4	157.5		0.9
	5″″	94.2	5.75 (d, 2.64)	1″″,3″″,4″″,6″″		5	94.5	5.69 ^d^ (d, 2.7)	−0.3
	6″″	153.8				6	153.7		0.1
H	1′′′′′	124.0			B	1′	124.3		−0.3
	2′′′′′,6′′′′′	152.2				2′,6′	152.1		0.1
	3′′′′′,5′′′′′	96.5	6.00 (s)	1′′′′′,2′′′′′,4′′′′′(H3′′′′′)		3′,5′	96.0	5.91 (s)	0.5
	4′′′′′	156.5				4′	157.9		−1.4

^a^ ^1^H NMR (600 MHz), ^13^C NMR (151 MHz), chemical shift (ppm), *J* (Hz), the signals of CHD_2_OD (δ_H_ 3.30) and ^13^CD_3_OD (δ_C_ 49.0) were used as internal references; ^b ^Correlations are from proton to carbon; ^c^ ND denotes not determined; ^d^ In ref. [17], the signals were oppositely assigned.

Since **1** was indicated to have 16 hydroxyl groups in the molecules as described above, a phenyl linkage should be present in the remaining part of **1** from **3**. The characteristic ^13^C signals observed at δ 102.4 and δ 102.6 in **1** were presumed to form a C–C bond between them. One set of 1H triplet ^1^H NMR signal at δ 5.91 (*J* = 2.06 Hz) coupling with 2H doublet signal at δ 5.96 (*J* = 2.06 Hz) assigned to the protons in the A or E rings in **3** was not shown in **1**. These data suggested that the part composed by the additional three aromatic rings (F, G, H rings) should be connected to the A or E rings of **3** by phenyl linkage in **1**.

The remaining part of **1** from **3** (composed by F, G, H rings) was suggested to have the structural feature of triphlorethol-B (**6**) (Figure 1), a reported phlorotannin from *E. cava* [17], by good agreement of the NMR data of A, F, G, H rings in **1** with those of C, D, A, B rings in fucodiphlorethol G (**5**) (Figure 1, Table 1). **5** is another reported phlorotannin [17] which contains the structure of **6**. The largest differences of ^1^H and ^13^C NMR chemical shifts between the F, G, H rings in **1**, and the D, A, B rings in **5**, respectively, were 0.13 ppm (^1^H) and 2.6 ppm (^13^C), respectively, as shown in Table 1. Therefore, **1** was suggested to have the structure whereby the carbon at the C2 position of the A or E rings of **3** forms a C–C bond with the carbon at the C2 position of the C ring (C2″) of **6**. The possibility that the C4 position, instead of the C2 position, in the A or E rings in **1** forms a C–C bond with the F ring in **1** can be ruled out for the following reason: A or E rings would be symmetric in this case, but the rings which form the C–C bond (A and F rings) in **1** were suggested to be asymmetrically substituted by the NMR data of A and F rings showing six ^13^C signals at the different chemical shifts and two doublet ^1^H signals at the different chemical shifts coupling with each other (see Table 1). 

Finally, it should be determined which ring A or E of the PFF-A (**3**) moiety is connected to the C ring of triphlorethol-B (**6**), in **1**. In CD_3_OD, the assignment of NMR signals corresponding to those in the A and E rings in **3** are interchangeable with each other due to their similar substitutive features, because NOE was not observed between the protons in the different rings. However, these signals were distinguished and assigned in (CD_3_)_2_SO in a previously published paper [13]. Therefore, although the NMR signals of **1** were severely broadened in (CD_3_)_2_SO, ^1^H–^1^H COSY (see Supplementary Figures S7–S9), HSQC and HMBC spectra of **1** were measured in (CD_3_)_2_SO to find the ^1^H and ^13^C signals corresponding to 2′/6′ and 4′ in the A ring or 2″/6″ and 4″ in the E ring by comparison with the previously reported data of **3** measured in (CD_3_)_2_SO [13]. Consequently, although the signal assignment was only partial, the ^13^C and ^1^H signals corresponding to 2″/6″ at δ 93.3, 5.71 (2H, d, *J* = 1.76 Hz) of the E ring of **3** were also shown at the same chemical shifts in **1**. Meanwhile, the signals corresponding to 2′/6′ (δ 93.5, 5.76 (2H, d, *J* = 1.76 Hz)) of the A ring of **3** were not found in **1**. The signals corresponding to 4″ and 4′ were shown at the same chemical shifts (δ 96.2, 5.82 (1H, brt)) in **3**. These data indicated that the A ring of **3**is connected to the C ring of **6** in **1** by phenyl linkage. Based on all of these data, the structure of **1** was determined as shown in Figure 1. 

### 2.4. Determination of the Structure of 974-B (**2**)

^1^H NMR spectrum of **2** seems to resemble that of **1** as shown in Supplementary Figure S2. Especially, the signals corresponding to triphlorethol-B (**6**) moiety in **1** were also present in **2**. Other signals except **6** moiety in **2** were almost identical to those in PFF-B (**4**) [16]. ^1^H and ^13^C NMR signals of **2** and **4** in CD_3_OD were assigned by analysis of 2D NMR (^1^H–^1^H COSY, HSQC, HMBC, see Supplementary Material) as listed in Table 2. The HMBC correlations observed in the B, C, D rings in **2** (measured setting *^n^J*_C,H_ 8 Hz) totally agreed with the reported data of **4** [16] including ^4^*J*_C,H_ correlations (Table 2). The largest difference of the ^13^C chemical shift between **2** and **4** in the A, B, C, D, E rings was 1.1 ppm, except for the signals of C1, C2, and C3 in the A or E rings of **2**. These data suggested that **2** contains the structure of **4**. The connectivity between the A and F rings in **2** was suggested to be same as that in **1**, because the chemical shifts of ^1^H and ^13^C NMR signals of A, E, F, G, and E rings in **2** were almost identical to those in **1**. Therefore, the structure of **2** was determined as that PFF-A (**3**) moiety in **1** was replaced by PFF-B (**4**) moiety as shown in Figure 1. 

**Figure 2:**
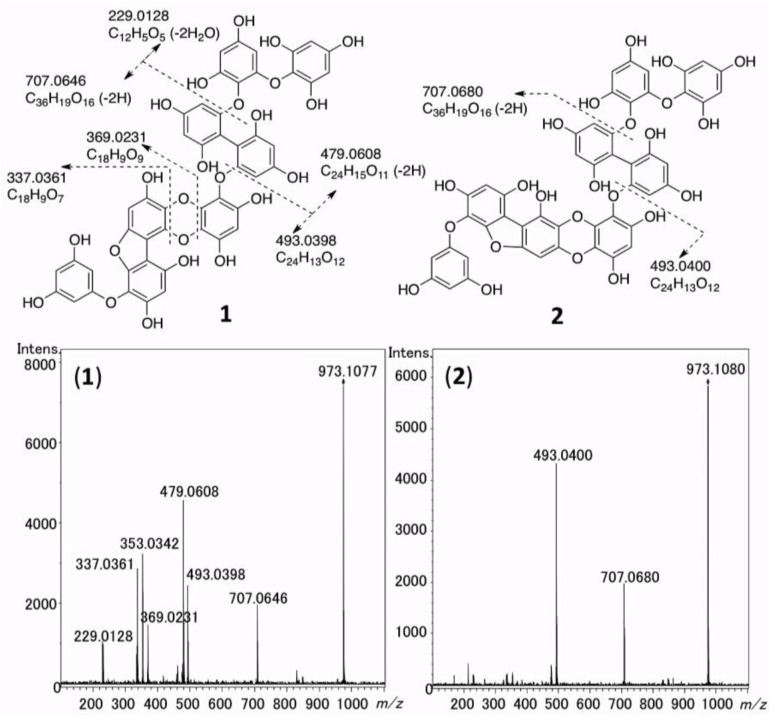
HR-ESI-MS/MS spectra of **1** and **2**, and interpretation of the fragment ions. HR-ESI-MS/MS spectra of **1** and **2** were measured by setting the [M−H]^−^ ions (**1**: *m/z* 973.1077, **2**: *m/z* 973.1080) as precursor ions in negative mode. The methanolic solution (1 μL) of purified **1** (2 ng) and **2** (2 ng) were applied to an ESI-Q-TOF MS spectrometer (see Section 4.4 for details).

### 2.5. The MS/MS Fragmentation of **1** and **2**

The HR-ESI-MS/MS spectra of **1** and **2** were measured to confirm the structures of **1** and **2**. As shown in Figure 2, from the molecular ions ([M − H]^−^) of **1** and **2**, the fragment ions corresponding to C_24_H_13_O_12_ (**1**: *m/z* 493.0398, **2**: *m/z* 493.0400, calcd 493.0407, Δ **1**: −0.9 mmu, **2**: −0.7 mmu) and C_36_H_19_O_16_ (**1**: *m/z* 707.0646, **2**: *m/z* 707.0680, calcd 707.0673, Δ **1**: −2.7 mmu, **2**: +0.7 mmu) were commonly shown. In addition, in the MS/MS spectrum of **1**, the fragment ions corresponding to C_24_H_15_O_11_ (*m/z* 479.0608, calcd 479.0614, Δ −0.6 mmu), C_18_H_9_O_9_ (*m/z* 369.0231, calcd 369.0247, Δ −1.6 mmu), C_18_H_9_O_8_ (*m/z* 353.0342, calcd 353.0297, Δ +4.5 mmu), C_18_H_9_O_7_ (*m/z* 337.0361, calcd 337.0348, Δ −1.3 mmu), and C_12_H_5_O_5_ (*m/z* 229.0128, calcd 229.0132, Δ −0.4 mmu) were detected. These fragment ions could be interpreted as shown in Figure 2, supporting the structures of **1** and **2** determined by NMR analysis as described above. It is notable that **1** was more liable to be fragmented than **2**, under the same condition. This difference should be due to the structural difference between **3** and **4** moieties in **1** and **2**, respectively.

### 2.6. DPPH Radical Scavenging Activity

**Figure 3:**
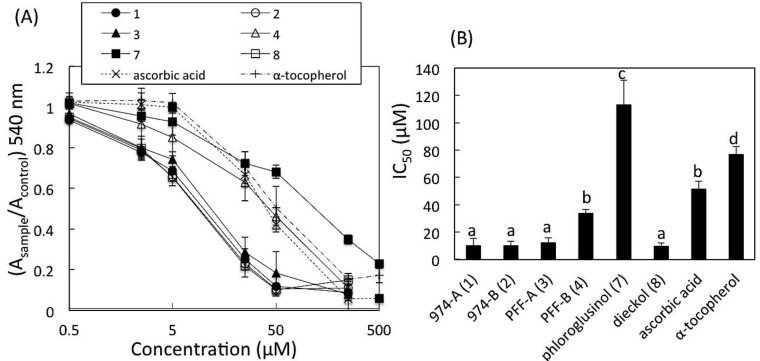
DPPH radical scavenging activities of **1**, **2**, **3**, **4**, **7**, and **8**. DPPH ethanolic solution (0.2 mM) was incubated with a sample (**1**, **2**, **3**, **4**, **7**, **8**, ascorbic acid, or α-tocopherol) for 30 min at 37 °C. (**A**) The ratio of absorbance at 540 nm in the presence of a sample (*A*_sample_) to that in the absence of a sample (*A*_control_) were plotted *vs*. concentrations of the sample (log). Data points are means ± SD (*n*=9–15). (**B**) The IC_50_ values of the samples obtained using Hill plots. Data points are means ± SD (*n*=3–5), and analyzed by a one-way ANOVA followed by the Tukey-Kramer test for multiple comparisons. a, b, c and d indicate statistically different groups (*p*<0.01, *p*<0.05, **3** *vs.***4**).

The DPPH radical scavenging activities of **1**, **2**, **3**, **4**, **7**, and **8** were determined and compared with those of DL-α-tocopherol and L-ascorbic acid as references. The results are shown in Figure 3. The IC_50_ values obtained from the result of three–five independent experiences using Hill plots were (mean ± SD, in μM): **1**, 10 ± 2.8 (*n* = 3); **2**, 11 ± 4.7 (*n* = 3); **3**, 12 ± 3.3 (*n* = 3); **4**, 34 ± 2.6 (*n* = 4); **7**, 110 ± 18 (*n* = 3); **8**, 10 ± 2.2 (*n* = 3); α-tocopherol, 77 ± 5.5 (*n* = 5); and L-ascorbic acid, 51 ± 5.5 (*n* = 3). By multiple comparisons, the values of IC_50_ of **1**, **2**, **3**, and **8** were significantly smaller than those of **4**, **7**, α-tocopherol and L-ascorbic acid (*p* < 0.01, *p* < 0.05, **3** *vs*. **4**), while the IC_50_ value of **4** was significantly smaller than only those of **7** and α-tocopherol (*p* < 0.01).

### 2.7. Intracellular Radical Scavenging Activity Measured Using DCFH-DA

**Figure 4:**
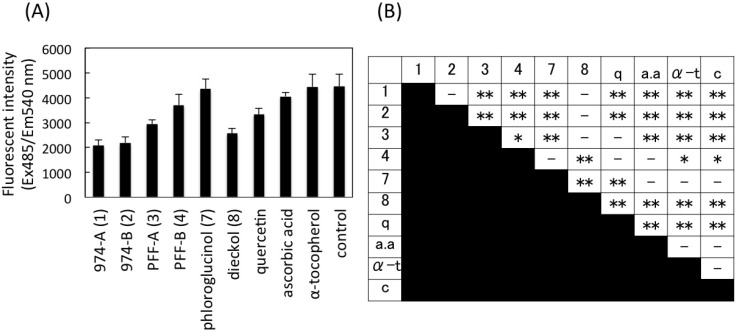
Intracellular radical scavenging activities of **1**, **2**, **3**, **4**, **7**, and **8**. (**A**) THP-1 macrophage-like cells were labeled with fluorescence dye, DCFH-DA (1 mM), for 30 min, and treated with 10 μM **1**, **2**, **3**, **4**, **7**, **8**, quercetin (q), ascorbic acid (a.a), and α-tocopherol (α-t) for 1 h. Fluorescence intensities of DCF (excitation 485 nm, emission 540 nm) due to deacethylation and oxidation of DCFH-DA by intracellular esterase and ROS were measured after stimulation with H_2_O_2_ (0.3 mM) for 2.5 h. Control (c) is antioxidant non-treated cells. Data points are means ± SD (*n*=5–6); (**B**) The results of data analysis by one-way ANOVA followed by the Tukey-Kramer test for multiple comparisons (**p<0.01, *p<0.05).

The intracellular radical scavenging activities of **1**, **2**, **3**, **4**, **7**, and **8** were measured using the cell-permeable and oxidation sensitive dye, 2′,7′-dichlorofluorescin diacetate (DCFH-DA), and compared with those of quercetin, ascorbic acid, and α-tocopherol. We chose this cell-based assay to evaluate the activities of these phlorotannins, because Li *et al.* [6] and Kang *et al.* [14,15], previously applied this assay to phlorotannins. DCFH-DA, originally non-fluorescent compound, is deacethylated by intracellular esterase, and then oxidized to highly fluorescent 2′,7′-dichlorofluorescein (DCF) by reactive oxygen species (ROS) [18]. Since Li *et al.* [6] used mouse macrophage-like cell line RAW264.7 for this assay, we used human macrophage-like cell line THP-1 (differentiated from lymphocyte-like phenotype) due to availability. Measurement of ROS in THP-1 macrophage by DCFH-DA assay has been reported by Estrella *et al.* [19]. Quercetin was also tested as a positive control, because Girard-Lalancette *et al*. [20] reported that quercetin showed positive activity by the DCFH-DA assay using L929 murine fibrosarcoma cell line. Initially, the absence of cytotoxicity of **1**, **2**, **3**, **4**, **7**, **8**, quercetin, ascorbic acid, and α-tocopherol to this cell line was confirmed at 15 μM by the same method as described in Section 2.7. To evaluate the intracellular radical scavenging activities, the fluorescence intensities of the cell treated with 10 μM antioxidants were measured and compared among them as shown in Figure 4. The findings showed that the fluorescence intensity of the cells treated with **1** and **2** were significantly smaller than those treated with other compounds (*p* < 0.01) except **8**. The ROS reducing activity of **3** was higher than that of **4** (*p* < 0.05), similarly to the result of DPPH assay (Figure 3). Quercetin decreased fluorescence compared with control and α-tocopherol (*p* < 0.01) as reported [20], but its activity was lower than those of **1**, **2**, and **8** (*p* < 0.01). The fluorescence of the cells treated with **7**, ascorbic acid, and α-tocopherol were not significantly different from that of control (*p* > 0.05). The data suggested that **1**, **2**, and **8** decreased intracellular production of ROS most significantly among the tested compounds.

### 2.8. Cell Viability

The effect of **1**, **2**, **3**, **4**, **7**, and **8** on the viability of THP-1 cell line (human macrophage-like phenotype differentiated from lymphocyte-like phenotype) was examined, related to the experiment in Section 2.7. The cells (5 × 10^4^ cells/well) were treated in the medium containing 15 μM of test compound and 0.1% ethanol for 24 h at 37 °C in a 96-well microtiter plate. The cell viability was measured using colorimetric assays using water-soluble tetrazolium salt, WST-8 [21]. The ratios of the viability in the presence of the test compound to that in the absence of test compound (only 0.1% ethanol, v/v, control) were obtained. As the result, the ratios (mean ± SD, *n* = 6) were 1.05 ± 0.07 (**1**), 1.12 ± 0.10 (**2**), 1.11 ± 0.11 (**3**), 1.13 ± 0.10 (**4**), 1.23 ± 0.12 (**7**), and 1.10 ± 0.11 (**8**). Only the ratio of **7** was significantly higher than control (*p* < 0.05), while the others were not significantly different from the control by a one-way ANOVA followed by the Tukey-Kramer test. Furthermore, the cell viabilities on mouse lymphoblast (P388) and mouse neuroblastoma (Neuro2A) (2.5 × 10^4^ cells/well) were also tested only for novel compounds, **1** and **2**, because other phlorotannins have been already used for many cell-based assays at higher concentration than 15 μM by other researchers (for example, [3,5,6,7,16]). The ratios of the viability of P388 in the presence of **1** or **2** at 15 μM to that in the absence of them (only 0.25% DMSO, v/v) after incubation for 24 h at 37 °C were 0.99 ± 0.11 and 1.10 ± 0.14, respectively (*n* = 3), and those ratios of Neuro2A were 0.99 ± 0.04 (**1**) and 1.00 ± 0.03 (**2**), respectively (*n* = 3). These data indicated that **1** and **2** have no effect on cell viability of these cell lines at 15 μM. 

## 3. Discussion

*E. kurome* is a common brown alga in Japan. Eckol, dieckol (**8**), 8,8′-bieckol and phlorofucofuroeckol-A (**3**) were previously isolated from this alga as the antiplasmin inhibitors, and their structures were determined by Fukuyama *et al.* [11,12,13]. The crude phlorotannins prepared from this alga was also reported to have bactericidal effects, more pronounced than those of the catechins [4]. Furthermore, it has recently been reported that the polyphenol powder prepared from this alga has inhibitory activities against α-amylase and α-glucosidase *in vitro*, and the positive effects on oral carbohydrate tolerance test *in vivo* in genetically diabetic KK-A^y^ mice [10]. In the present study, we succeeded to isolate and determine the structures of two novel phlorotannins, 974-A (**1**) and 974-B (**2**), from this *E. kurome* polyphenol powder, although we have not studied whether **1** and **2** have such antidiabetic activities *in vivo*. The isolated yield of the total of **1** and **2** from polyphenols in this source was estimated to be approximately 4% (w/w). We have also isolated **1** (approximately 1 mg) from the methanolic extract of another brown alga *Eisenia arborea* Areschoug (dry weight 300 g, collected in Shizuoka Prefecture, Japan, in August 2008) by almost the same method as described in this paper, and identified **1** by ESI-MS and NMR spectroscopy (not shown). Therefore, at least **1** is not the specific phlorotannin to *E. kurome*, although screening of **1** and **2** in other algae has not been performed yet. 

In the process of structural determination of **1** and **2**, broadening of the NMR signals in (CD_3_)_2_SO was the most difficult problem to be solved, probably due to the large size of the molecules. It was much improved in CD_3_OD, but no NOE correlation between the protons in the different rings was observed in CD_3_OD. This hampered determination of the connectivity between the rings. To solve this problem, we first attempted methylation of **2** by trimethylsilyldiazomethane. However, even for methylated **2**, clear and informative NOEs were not observed. Thus, we ultimately determined the structures of **1** and **2** by comparison with the NMR data of previously reported phlorotannins, **3**, **4**, **5** and **6**. The elucidated structures of **1** and **2** were supported by the fragmentation patterns shown in their HR-MS/MS spectra.

In this study, DPPH radical scavenging assay was chosen as one of the methods to evaluate *in vitro* antioxidant activities of the new phlorotannins **1** and **2**, because this assay is commonly used in plant and food chemistry [1]. Furthermore, according to the data reported by Li *et al.* [6], the activities determined by this assay seemed to be almost parallel to the results obtained by other radical scavenging assays, such as hydroxyl radical, superoxide anion radical, and peroxyl radical. However, the lower steric accessibility of DPPH radical to the antioxidants would be suspected, and Gülcin [22] pointed out that many large antioxidant compounds that react quickly with ROO^-^ may react slowly or may even be inert in DPPH assay. In the present study, we used only purified compounds for the DPPH assay, not containing ROO^-^ species, so the latter concern could be avoided even for the large molecules, **1** and **2** (MW 974). In the present results, **1**, **2**, **3** and **8** showed significantly higher potencies than **4**, even though **3** and **4** are isomers with each other of which structural difference between them is only the substituted position in the C ring. This is the first data to directly compare the DPPH radical scavenging activities between **3** and **4**, although the activities of **3** and **4** were independently reported. The activity of **3** was reported by Li *et al.* [6] and Shibata *et al.* [23], and the activity of **4** was reported by Lee *et al.* [24]. For the structure-activities relationship, Li *et al.* [6] proposed that the numbers of hydroxyl groups in phlorotannins are responsible to the antioxidant activity. The numbers of hydroxyl groups in the phlorotannins tested in this study were: **1** and **2** (OH-16), **3** and **4** (OH-9), and **8** (OH-11), while the activities were **1**, **2**, **3**, **8** > **4**. Based on this data, the activity might be thought to depend on the structure (substitution pattern) of phlorotannins.

Furthermore, the intracellular radical scavenging activity of these phlorotannins was evaluated using DCFH-DA and THP-1 macrophage-like cell line. The result indicated that **1**, **2**, and **8** decrease the intracellular ROS most strongly among the tested compounds, and positive effects of **3** and **4** were also detected, while such activity was not detected for **7** under the condition in this study. The order of the activities was **1**, **2**, **8** ≥ **3** > **4** > **7**, almost similar to that of DPPF assay. Li *et al.* [6] also performed a similar DCFH-DA assay for phlorotannins using RAW264.7 cell line and showed that all the phlorotannins tested, including **3**, **7**, and **8**, had positive activity, but the activity of **7** was the weakest among them. Our result seems to concur with the result of Li *et al.* [6]. Such activities of ascorbic acid and α-tocopherol were not detected in this study. Girard-Lalancette *et al.* [20] also reported that activity of α-tocopherol is much lower than those of some catechins, such as caffeic acid, gallic acid, and quercetin by the DCFH-DA assay using the L929 murine fibrosarcoma cell line. For this reason, quercetin was used as the positive control in our present study, and showed the activity at the same level as **3** and **4**. 

Absence of cytotoxicity of **1**, **2**, **3**, **4**, **7**, and **8** (15 μM) to THP-1 cell (macrophage-like form) was confirmed. Furthermore, absence of cytotoxicity of **1** and **2** (15 μM) to two mouse tumor cell lines (Neuro2A, P388) were also confirmed. We suppose that low cytotoxicity of **1** and **2** is an advantage as potential natural antioxidants. Although we have not directly studied whether **1** and **2** could be cell-permeable and effectively act in intracellular environment, Teng *et al.* [25] recently reported that small polyphenols could be absorbed rapidly by Caco-2 cells probably *via* transporters. Also, in the above DCFH-DA assay in this study, **1** and **2** were indicated to reduce the intracellular ROS. Based on these data, we cannot exclude the possibility that phlorotannins with large molecular sizes (or their degraded forms) could be permeable to cells and act intracellularly. Further biological activities of **1** and **2**, such as inhibition of specific enzymes and effects on cellular functions, are now in progress. 

## 4. Experimental Section

### 4.1. General Materials

^1^H NMR (600 MHz) and ^13^C NMR (151 MHz) spectra were recorded on an Agilent 600 MHz NMR spectrometer (Agilent Technologies, Santa Clara, CA, USA) in CD_3_OD and (CD_3_)_2_SO. The signals of CHD_2_OD (δ_H_ 3.30) and ^13^CD_3_OD (δ_C_ 49.0), and (CHD_2_)_2_SO (δ_H_ 2.50) and (^13^CD_3_)_2_SO (δ_C_ 39.5) were used as internal references. HMBC spectra were measured setting *^n^J*_C,H_ 8 Hz. ESI-MS spectra (conventional) were obtained on API2000 mass spectrometer (AB Sciex, Foster City, CA, USA). HR-ESI-TOF-MS spectra and HR-ESI-MS/MS (Q-TOF) spectra were recorded on MicrOTOF-QII (Bruker Daltonics, Bremen, Germany). Fast atom bombardment (FAB)-MS spectra were recorded on JEOL JMS700 MS Station (Akishima, Japan). 

DL-α-tocopherol and hydrogen oxide were purchased from Wako Chemical Industries (Osaka, Japan), L-ascorbic acid was purchased from Nacalai tesque (Kyoto, Japan), phlorogrucinol, acetic anhydride, Hank’s balanced salt solution (HBSS), and 2′,7′-dichlorofluorescin diacetate (DCFH-DA) were purchased from Sigma-Aldrich Chemical Co. (St. Louis, MO, USA), and DPPH was purchased from Tokyo Chemical Industry Co., Ltd. (Tokyo, Japan), and quercetin was purchased from Cayman Chemical. Other reagents were special grade and purchased from Wako Chemical Industries. 

### 4.2. Isolation of Phlorotannins

*Ecklonia kurome* Okamura, collected from Kyoto Prefecture, Japan, from May to June in 2009, was dried for one or two days in the beach side. Then, the alga was smashed into a powder (particle size, less than 335 μm). The powdered *E. kurome* was extracted with water/ethanol (3:7, v/v, 10 volumes of the algal powder) for 24 h at room temperature with occasional (a few times) agitation, and then filtered. The extract was concentrated under reduced pressure and adsorbed onto Diaion HP-20 (Mitsubishi Chemical Co., Tokyo, Japan). Subsequently, the resin was washed with distilled water, and then the crude polyphenol was eluted with water/ethanol (4:6, v/v). After the solvent in the eluent was evaporated, the residue was freeze-dried to yield crude polyphenol powder (yield, 6.0%) [10]. The total polyphenol content of this polyphenol power was previously reported to be over 70% [10]. 

This crude polyphenol powder (500 mg) dissolved in 0.9 mL of water/methanol (2:7, v/v) was subjected to reversed phase liquid chromatography on a Cosmosil 75C_18_-OPN (Nacalai tesque, Kyoto, Japan) packed in a glass column (10 i.d. × 250 mm), equilibrated with water, and a stepwise elution (water/methanol 10:0, 3:1, 1:1, 0:10 (v/v), 150 mL for each, flow rate 1.0 mL/min) was employed. Elution of phlorotannins was monitored by ESI-MS (conventional) analysis in negative-mode. An aliquot of each fraction of the eluate from the column was applied to ESI-MS from the sample injector, flowing methanol with the flow rate at 0.2 mL/min from the pump (flow injection mode). A part of water-methanol (1:1, v/v) elution showed the ions corresponding to [M − H]^−^ *m/z* 601, 741 and 973 in ESI-MS spectra, indicating the presence of **1**, **2**, **3**, **4**, and **8**. This fraction was concentrated using rotary evaporator (dried weight, 80 mg), divided into three portions, and each portion was applied to another reversed phase column, a Mightysil RP-18 GPII (10 i.d. × 250 mm, 5 μm, Kanto Chemical, Co., Inc., Tokyo, Japan), equilibrated with formic acid/methanol/water (1:40:59, v/v, flow rate 0.5 mL/min) at 30 °C. Elution of phlorotannins with the same solvent was monitored by a Hitachi diode array detector L-7455 (190–500 nm, phlorotannins were detected as the peaks showing λ_max_ 204–208 nm under this condition) and ESI-MS analysis. Dieckol (**8**) (5 mg), 974-B (**2**) (9 mg) and 974-A (**1**) (6 mg) were mainly eluted in 90–100 mL, 105–120 mL and 130–140 mL fractions, respectively, in almost pure forms. Further, PFF-B (**4**) (1 mg) and PFF-A (**3**) (4 mg) were mainly eluted in 140–150 mL and 155–165 mL, respectively (partially overlapped with **1**). **1**, **2**, **3**, **4**, and **8** were obtained as light brown powder. **3** [13], **4** [16] and **8** [12] were identified by comparison with previously reported spectral data: ESI-MS, [M − H]^−^ *m/z* 601 (**3**, **4**) and 741 (**8**); NMR data of **3** and **4** in CD_3_OD are shown in Table 1 and Table 2, respectively. HR-ESI-TOF MS, **1**: [M − H]^−^ *m/z* 973.1069, calcd for C_48_H_29_O_23_ 973.1100, Δ −3.1 mmu,** 2**: [M − H]^−^ *m/z* 973.1062, calcd for C_48_H_29_O_23_ 973.1100, Δ −3.8 mmu (see Supplementary Material).

### 4.3. Acetylation of **1** and **2**

**2** (2.2 mg) in the mixture of acetic anhydride (300 μL) and dehydrated pyridine (600 μL) was allowed to stand for 18.5 h at room temperature [26] (other reaction time was never tested). After the solvent was evaporated under reduced pressure, the residue was partitioned between ethyl acetate and water. The ethyl acetate layer was dried by the stream of N_2_ gas and dissolved in acetonitrile. The solution was subjected to reversed phase HPLC purification on a Mightysil RP-18 GP II (4.6 i.d. × 250 mm) equilibrated with formic acid/acetonitrile/water (1:30:69, v/v) at 30 °C. Then, acethylated 974-B (**2a**) was eluted with acid/acetonitrile/water (1:80:19, v/v, flow rate 0.5 mL/min). Elution was monitored by a Hitachi diode array detector L-7455. Acethylated 974-B (**2a**): HR-FAB-MS ([M + H]^+^ *m/z* 1647.2947, calcd for C_80_H_63_O_39_ 1647.2947, Δ +0.0 mmu). Acethylation for **1** was simplified as follows. **1** (10 μg) was acethylated in the mixture of acetic anhydride (30 μL) and dehydrated pyridine (60 μL) as described above. Then, the mixture was partitioned between ethyl acetate and water. A part of the ethyl acetate layer was applied to HR-ESI-TOF MS in flow injection mode (see, Section 4.2) with methanol. Acethylated 974-A (**1a**): ([M + Na]^+^ *m/z* 1669.2770, calcd for C_80_H_62_NaO_39_ 1669.2760, Δ +1.0 mmu).

### 4.4. HR-ESI-MS/MS Measurement

HR-ESI-MS/MS (negative) spectra of **1** and **2** were measured in flow injection mode (see Section 4.2) with methanol (flow rate 0.2 mL/min) using autoMS/MS mode on MicrOTOF-QII MS spectrometer. The parameters of mass spectrometer were set as following: nebulizer gas 1.6 Bar, capillary 4000 V, dry heater 180 °C, end plate offset −500 V, dry gas 8.0 L/min, collision cell RF 650 Vpp, collision energy 24.6–49.2 eV (sweeping mode). The [M − H]^−^ of **1** and **2**, *m/z* 973.1100 ± 0.5, were set as the precursor ions. The methanolic solution (1 μL) of purified **1** (2 ng) and **2** (2 ng) were applied to the spectrometer using an autosampler (Shimadzu SIL-30AC) and an LC-pump (Shimadzu LC-30AD).

### 4.5. DPPH Radical Scavenging Activity

**1**, **2**, **3**, **4, **and **8** were isolated from *E. kurome* as described above, and quantified by weighting after completely drying under low pressure, and also by ^1^H NMR using the ratio of the integration values of ^1^H signals of the compounds and the signal of CHD_2_OD [27]. DPPH radical scavenging assay was carried out using a flat-bottom 96-well microtiter plate (TPP Techno Plastic Products AG. Transadingen, Switzerland) as described by Nanjo *et al*. [28], and Kimura *et al.* [29] with modifications. Briefly, a 5 μL of sample solution (**1**–**4**, **7**, **8**, and DL-α-tocopherol in ethanol, and L-ascorbic acid in water) was mixed with 95 μL of the 1:1 (v/v) mixture of 0.4 mM ethanolic solution of DPPH and 100 mM aqueous MES (sodium 2-(*N*-morpholino)ethanesulfonate) buffer (pH 6.0) in a well using a plate mixer for 1 min. After incubation for 30 min at 37 °C in the dark, absorbance of each well was measured at 540 nm setting the reference at 650 nm using a microplate reader (Infinite F200 Pro, TECAN, Männedorf, Switzerland). Absorbance was measured at 540 nm instead of 517 nm (λ_max_ of DPPH radical) just for the instrumental reason of our microplate reader. It was confirmed that statistic difference was not detected between the IC_50_ values of α-tocopherol determined at 540 nm and that at 510 nm (only 510 nm filter was available for this test). The final concentrations of the compounds were at the range of 0.5–500 μM. The radical scavenging activity of the samples was calculated as a ratio of remaining free radical of DPPH according to *A*_sample_/*A*_control_, where *A*_control_ is the absorbance of DPPH incubated without test compound, and *A*_sample_ is the absorbance of DPPH incubated with test compound. All experiments were carried out in triplicate, with each concentration tripled, except for α-tocopherol (five times) and **4** (four times). The IC_50_ values, obtained using Hill plots from three independent experiments, were statistically compared among all the tested compounds by a one-way ANOVA, followed by the Tukey-Kramer test for multiple comparisons.

### 4.6. Cellular Radical Scavenging Activity Measured Using DCFH-DA

The cellular radical scavenging activities of phlorotannins were estimated by the method using DCFH-DA by following the report by Li *et al.* [6] with modification. THP-1 (human lymphocyte-like cell) cell line was provided by RIKEN BRC (Tsukuba, Japan) through the National Bio-Resource Project of the MEXT, Japan, and cultured in RPMI1640 (Sigma), supplemented with 10% (v/v) fetal bovine serum (Biowest, Nuaillé, France) and 1% (v/v) Penicillin Streptomycin (10,000 units/mL Penicillin, 10,000 μg/mL Streptomycin GIBCO) at 5% CO_2_ and 37 °C. The cells were inoculated at density of 5 × 10^4^ cells/well and differentiated into macrophage-like phenotypes by incubation with 40 nM of phorbol-12-myristate-13-acetate (PMA) for 48–72 h [30] in microtiter 96-well plates. After washing with Hank’s balanced salt solution (HBSS), the cells were labeled by incubation with 1 mM DCFH-DA in HBSS for 30 min, and washed with HBSS three times. For preparation of 1 mM DCFH-DA in HBSS solution, DCFH-DA was initially dissolved in ethanol (16 mg/mL), and then this solution was diluted with HBSS warmed at 37 °C to solubilize DCFH-DA completely. Cells were then treated with 10 μM of tested phlorotannins, quercetin, ascorbic acid, and α-tocopherol in HBSS for 1 h. After washing with HBSS, 300 μM of H_2_O_2_ in HBSS was added to cells. All these steps were performed under dark condition. The formation of 2′,7′-dichlorofluorescein (DCF) from DCFH-DA, due to deacetylation by intracellular esterase and oxidation in the presence of ROS, was read after 2.5 h incubation in the dark at the excitation wavelength (Ex) of 485 nm and the emission wavelength (Em) of 540 nm using a fluorescence microplate reader (Infinite F200 Pro, TECAN). As the control, the cells were only treated with H_2_O_2_, and not treated with antioxidant. Six wells were measured for each compound, and the experiments were tripled. The fluorescent intensity was statistically compared among tested compounds by a one-way ANOVA, followed by the Turkey-Kramer test for multiple comparisons.

### 4.7. Cell Viability

THP-1 (see, Section 4.6), P388 (mouse lymphoblast-like cell) and Neuro2A (mouse neuroblastoma cell) cell lines were provided by RIKEN BRC (Tsukuba, Japan) through the National Bio-Resource Project of the MEXT, Japan, and cultured in RPMI1640 (Sigma), supplemented with 10% fetal bovine serum (Biowest, Nuaillé, France) and 1% Penicillin Streptomycin (10,000 units/mL Penicillin, 10,000 μg/mL Streptomycin GIBCO) at 5% CO_2_ and 37 °C. Cytotoxic effects were evaluated using WST-8 (2-(2-methoxy-4-nitrophenyl)-3-(4-nitrophenyl)-5-(2,4-disulfophenyl)-2*H*-tetrazolium, monosodium salt) assay [19]. THP-1 cells (lymphocyte-like form) were inoculated at density of 5 × 10^4^ cells/100 μL/well into 96-well plates and differentiated into macrophage-like phenotypes by incubation with 40 nM of PMA containing medium [30] for 72 h. Then, THP-1 macrophage-like cells were incubated in the medium containing 15 μM of **1**, **2**, **3**, **4**, **7**, and **8**, and 0.1% ethanol (v/v) for 24 h. P388 and Neuro2A were inoculated at density of 2.5 × 10^4^ cells/100 μL/well into 96-well plates and treated with 15 μM of **1** or **2** and 0.25% of DMSO (v/v). After a 24-h incubation, 5 μL of WST-8 (Cell Counting Kit-8, Dojindo, Kumamoto, Japan) was added to the cells and then further incubated for 2.5 h. The absorbance at 450 nm was measured, setting the reference at 655 nm, using a microplate reader (Model 680, Bio Rad, Hercules, CA, USA). For control, the cells were also incubated in a medium containing 0.1% ethanol (THP-1) or 0.25% of DMSO (P388 and Neuro2A) in the absence of phlorotannins.

## 5. Conclusions

Two novel phlorotannins (molecular weight 974) temporarily named 974-A and 974-B, were isolated from the edible brown alga *Ecklonia kurome*, and their structures were determined mainly by NMR analysis and supported by HR-MS/MS data. They showed DPPH radical scavenging activities as potent as dieckol and phlorofucofuroeckol-A, and more potent than phlorofucofuroeckol-B, phloroglucinol, α-tocopherol, and ascorbic acid. They also showed significantly higher intracellular radical scavenging activity than phlorofucofuroeckol-A, phlorofucofuroeckol-B, and quercetin using DCFH-DA dye. The viability of three tumor cell lines was not affected by 974-A and 974-B at the minimum of 15 μM, indicating their potency as natural antioxidants.

## Supplementary Files

Supplementary File 1 Supplementary Information (PDF, 1119 KB)
