# Supplementary material for: Isolation and Structural Determination of Two Novel Phlorotannins from the Brown Alga Ecklonia kurome Okamura, and Their Radical Scavenging Activities
Source: Mar Drugs. 2013 Jan 18;11(1):165–83. doi: 10.3390/md11010165 (PMC3564165; doi:10.3390/md11010165)

Supplementary Information

Figure S1. The HR ESI-MS spectra of **1** and **2**. The methanolic solutions (1 μL) of purified **1** (1.0 ng) (**A**) and **2** (0.8 ng) (**B**) were applied to an ESI-TOF-MS in negative mode. 2

Figure S2. ^{1}H NMR spectra of **1**, **2**, **3** and **4** (600 MHz, CD_3OD, $C\underline{H}D_2OD$ 3.30 ppm). 2

Figure S3. ^{13}C NMR spectrum of **1** in CD_3OD (151 MHz, $^{13}CD_3OD$ 49.0 ppm). 3

Figure S4. COSY spectrum of **1** (600 MHz, CD_3OD, $C\underline{H}D_2OD$ 3.30 ppm). 3

Figure S5. HSQC spectrum of **1** (600 MHz, CD_3OD, $C\underline{H}D_2OD$ 3.30 ppm, 151 MHz, $^{13}CD_3OD$ 49.0 ppm). 4

Figure S6. HMBC spectrum of **1** (600 MHz, CD_3OD, $C\underline{H}D_2OD$ 3.30 ppm, 151 MHz, $^{13}CD_3OD$ 49.0 ppm, $^{n}J_{C,H}$ 8 Hz). 4

Figure S7. ^{1}H NMR spectrum of **1** in $(CD_3)_2SO$ (600 MHz, $(C\underline{H}D_2)_2SO$ 2.50 ppm). 5

Figure S8. ^{1}H NMR spectrum of **1** in $(CD_3)_2SO$ (600 MHz, $(C\underline{H}D_2)_2SO$ 2.50 ppm) (peak label: chemical shifts in Hz). 5

Figure S9. COSY spectrum of **1** in $(CD_3)_2SO$ (600 MHz, $(C\underline{H}D_2)_2SO$ 2.50 ppm). 6

Figure S10. ^{13}C NMR spectrum of **2** in CD_3OD. (151 MHz, $^{13}CD_3OD$ 49.0 ppm). 6

Figure S11. COSY spectrum of **2** (600 MHz, CD_3OD, $C\underline{H}D_2OD$ 3.30 ppm). 7

Figure S12. HSQC spectrum of **2** (600 MHz, CD_3OD, $C\underline{H}D_2OD$ 3.30 ppm, 151 MHz, $^{13}CD_3OD$ 49.0 ppm). 7

Figure S13. HMBC spectrum of **2** (600 MHz, CD_3OD, $C\underline{H}D_2OD$ 3.30 ppm, 151 MHz, $^{13}CD_3OD$ 49.0 ppm, $^{n}J_{C,H}$ 8 Hz). 8

Figure S14. ^{1}H NMR spectrum of **2** in $(CD_3)_2SO$ (600 MHz, $(C\underline{H}D_2)_2SO$ 2.50 ppm). 8

Figure S1. The HR ESI-MS spectra of **1** and **2**. The methanolic solutions (1 μL) of purified **1** (1.0 ng) (**A**) and **2** (0.8 ng) (**B**) were applied to an ESI-TOF-MS in negative mode.

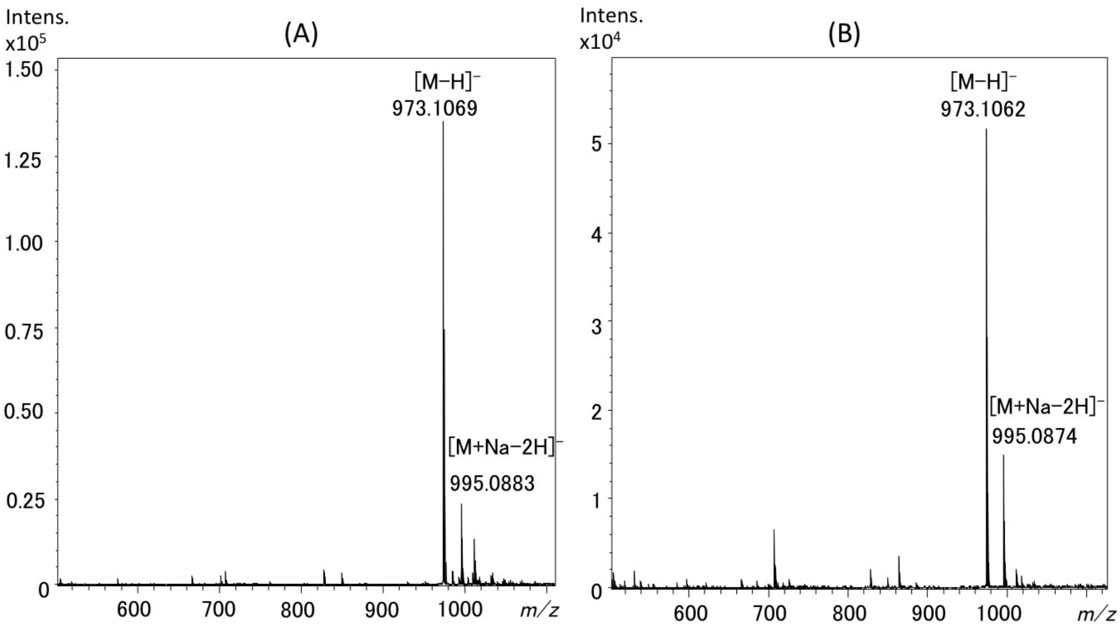

Figure S2. ^{1}H NMR spectra of **1**, **2**, **3** and **4** (600 MHz, CD_3OD, C<u>H</u>D_2OD 3.30 ppm).

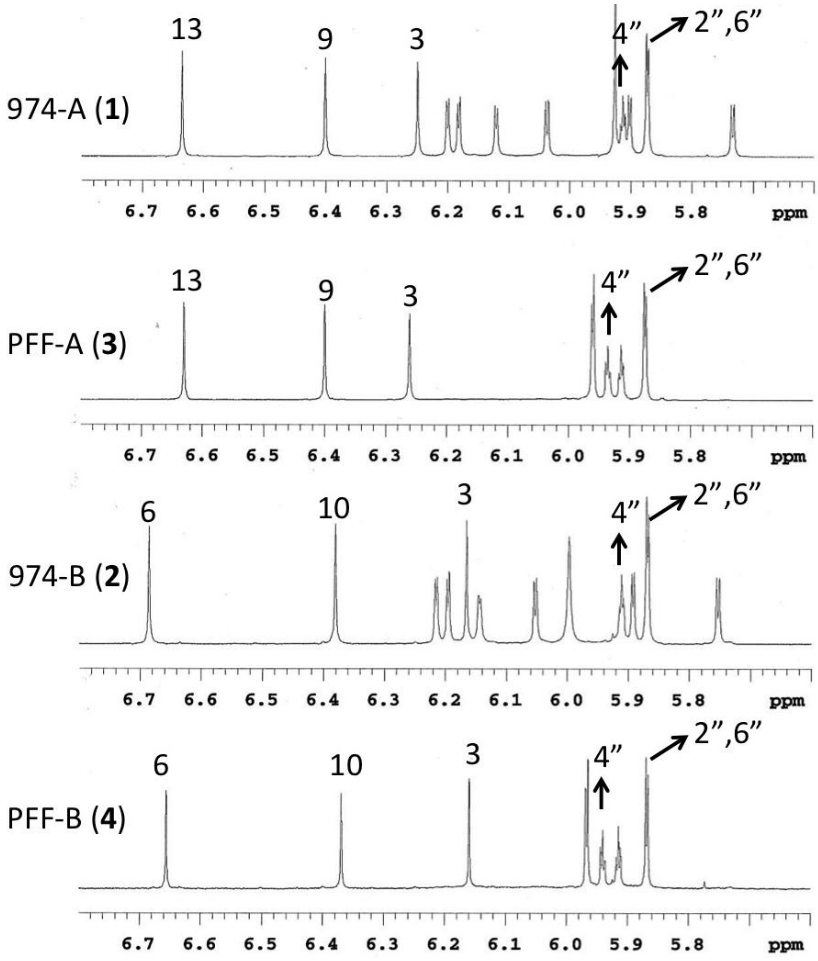

Figure S3. ^{13}C NMR spectrum of **1** in CD_3OD. (151 MHz, $^{13}CD_3OD$ 49.0 ppm).

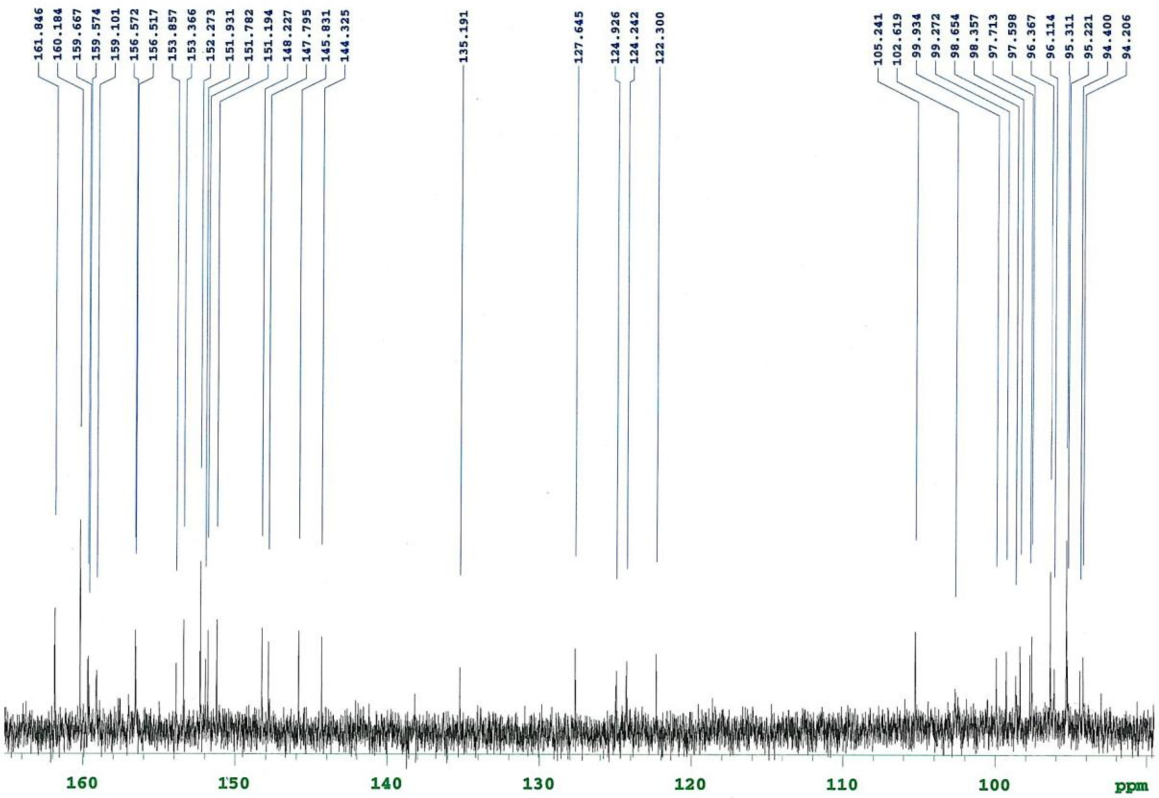

Figure S4. COSY spectrum of **1** (600 MHz, CD_3OD, $C\underline{H}D_2OD$ 3.30 ppm).

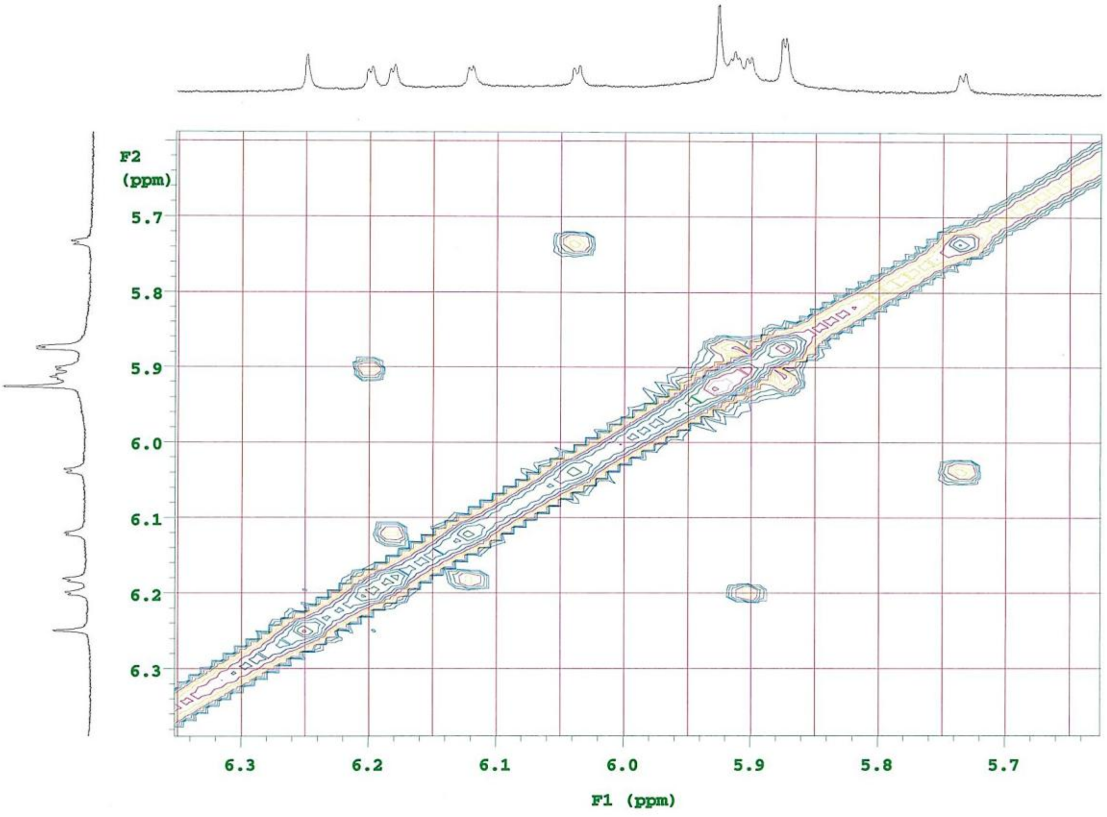

Figure S5. HSQC spectrum of **1** (600 MHz, CD_3OD, C<u>H</u>D_2OD 3.30 ppm, 151 MHz, $^{13}CD_3OD$ 49.0 ppm).

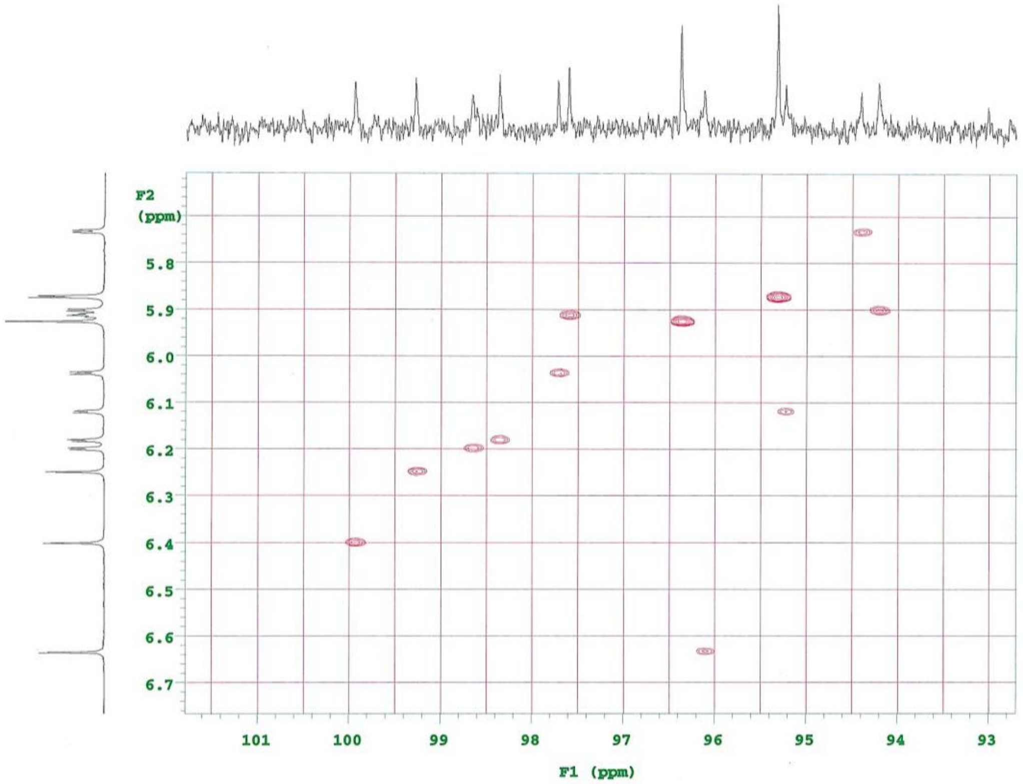

Figure S6. HMBC spectrum of **1** (600 MHz, CD_3OD, CHD_2OD 3.30 ppm, 151 MHz, $^{13}CD_3OD$ 49.0 ppm, $^{n}J_{C,H}$ 8 Hz).

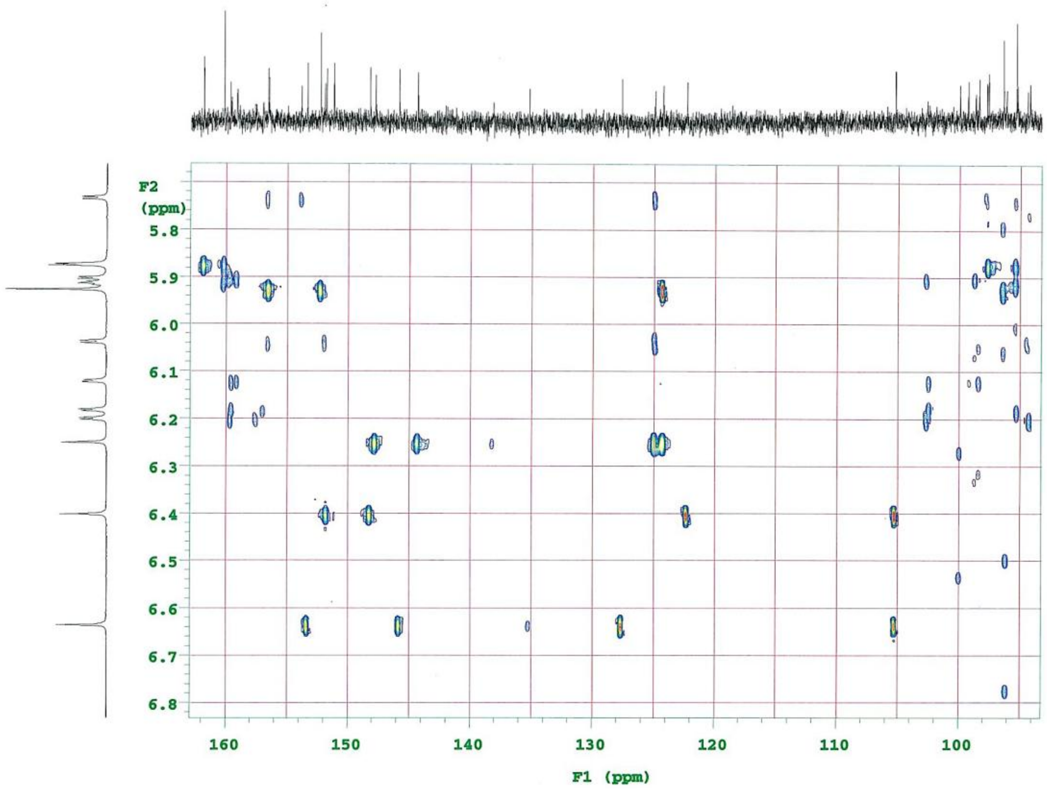

Figure S7. ^{1}H NMR spectrum of **1** in $(CD_3)_2SO$ (600 MHz, $(C\underline{H}D_2)_2SO$ 2.50 ppm).

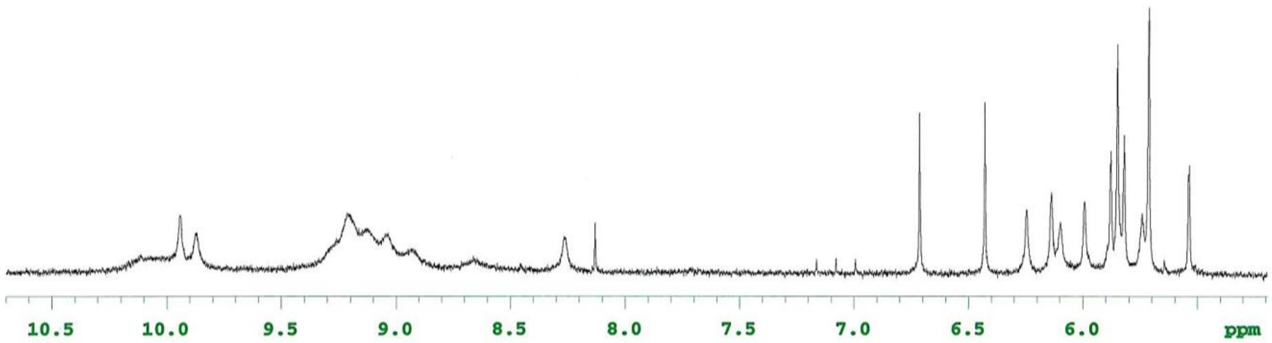

Figure S8. ^{1}H NMR spectrum of **1** in $(CD_3)_2SO$ (600 MHz, $(C\underline{H}D_2)_2SO$ 2.50 ppm) (peak label: chemical shifts in Hz).

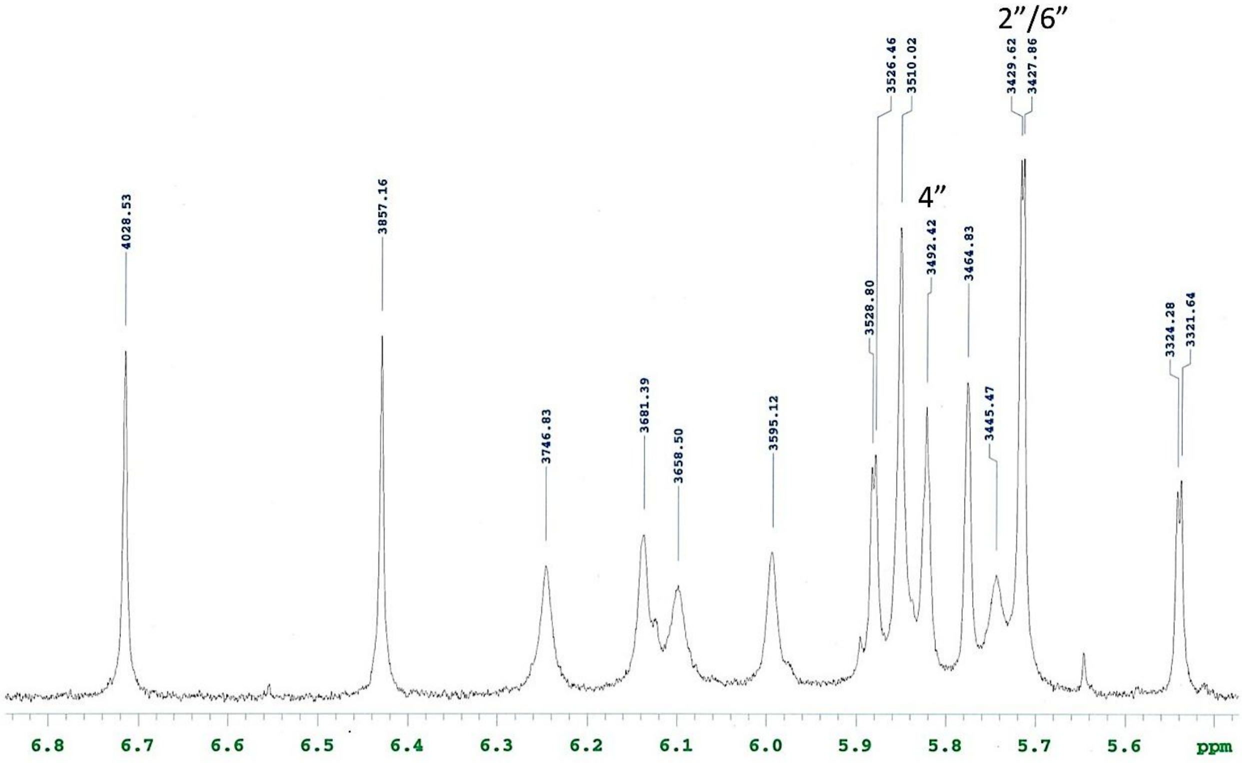

Figure S9. COSY spectrum of **1** in $(CD_3)_2SO$ (600 MHz, $(C\underline{H}D_2)_2SO$ 2.50 ppm).

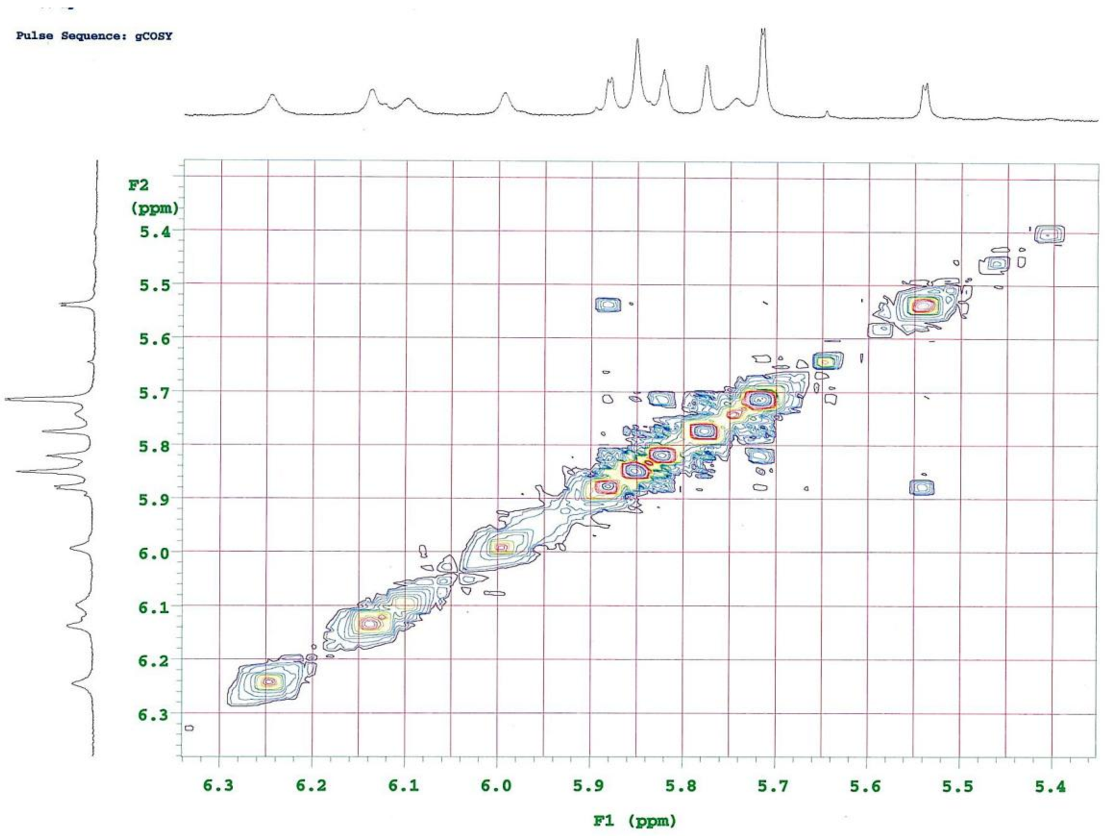

Figure S10. ^{13}C NMR spectrum of **2** in CD_3OD. (151 MHz, $^{13}CD_3OD$ 49.0 ppm).

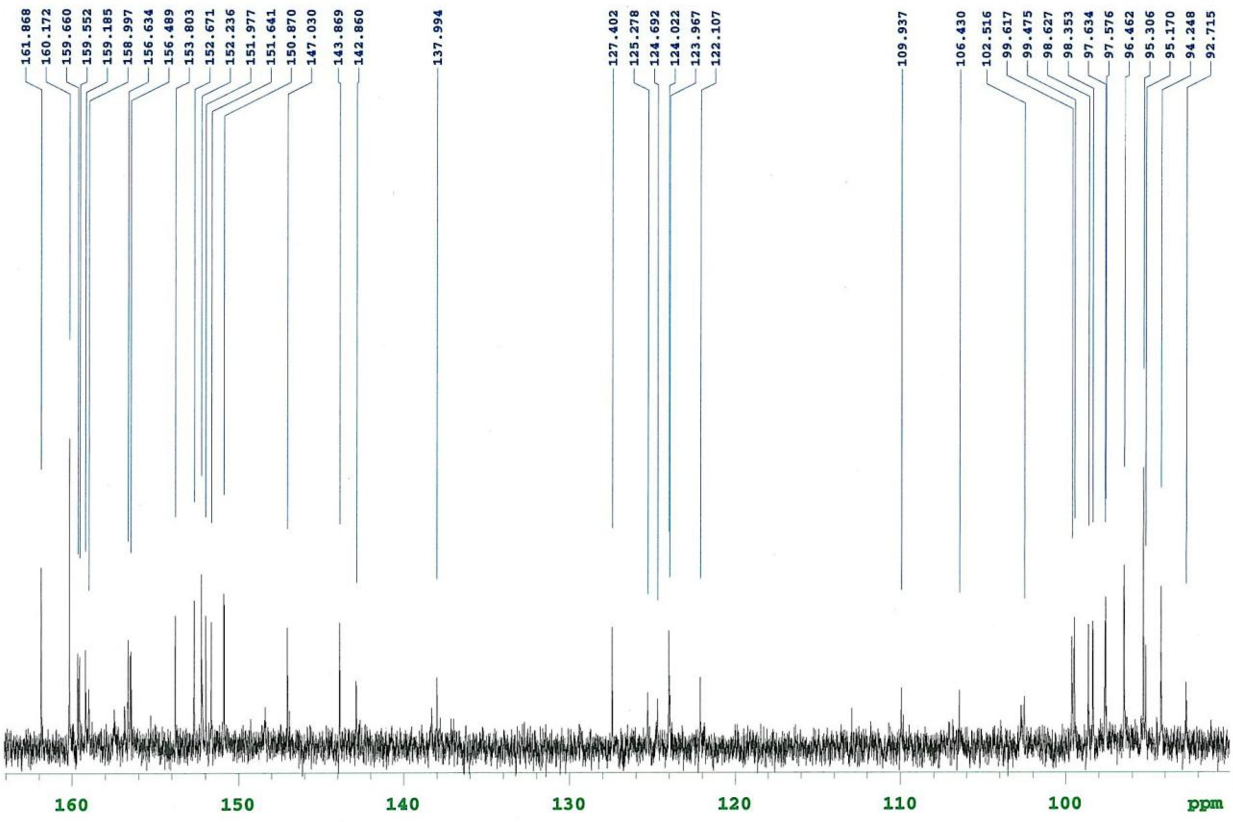

Figure S11. COSY spectrum of **2** (600 MHz, CD_3OD, $C\underline{H}D_2OD$ 3.30 ppm).

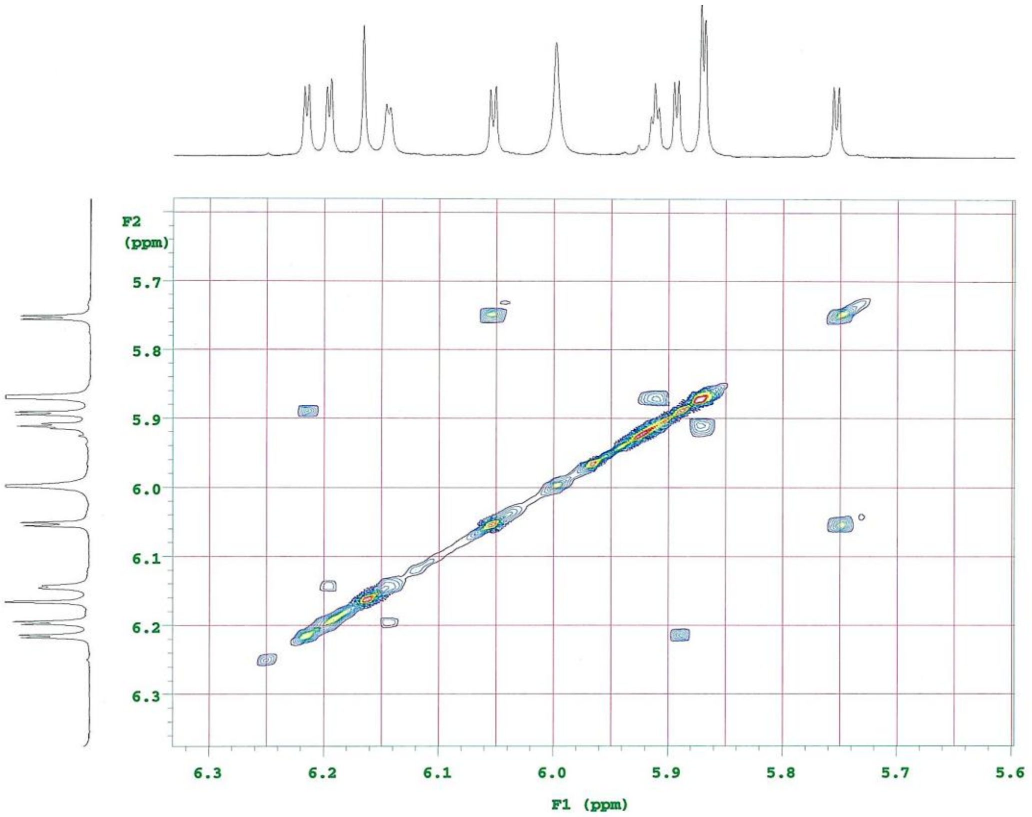

Figure S12. HSQC spectrum of **2** (600 MHz, CD_3OD, $C\underline{H}D_2OD$ 3.30 ppm, 151 MHz, $^{13}CD_3OD$ 49.0 ppm).

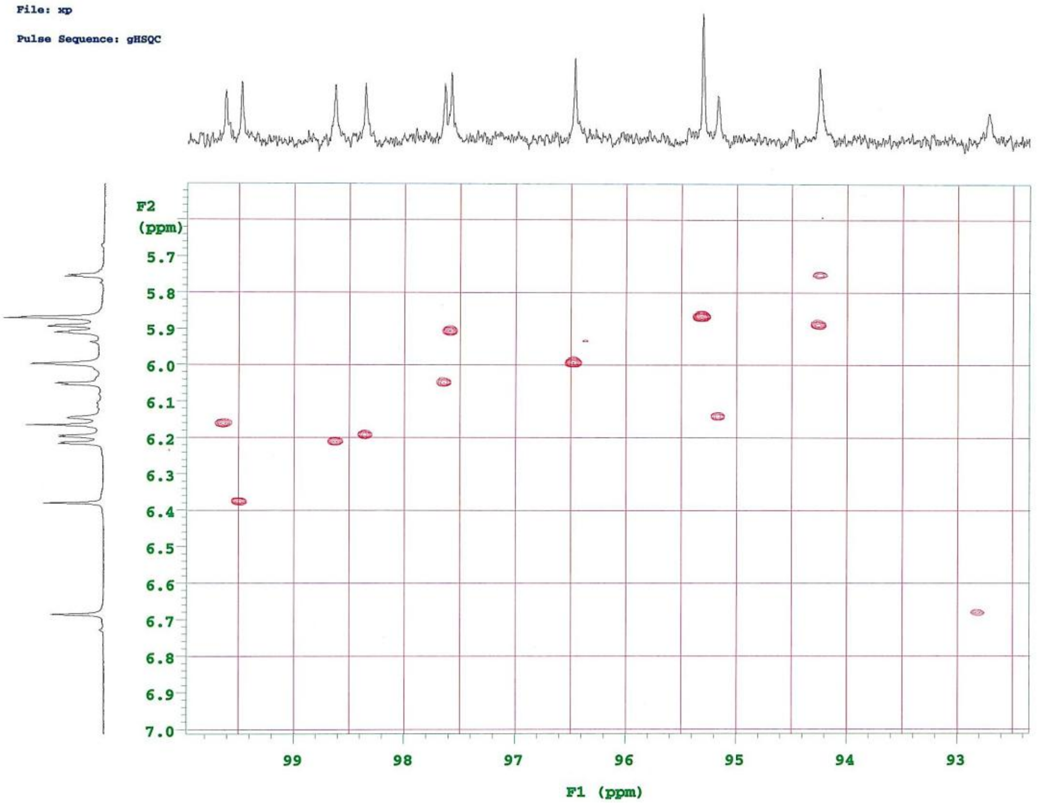

Figure S13. HMBC spectrum of **2** (600 MHz, CD_3OD, $C\underline{H}D_2OD$ 3.30 ppm, 151 MHz, $^{13}CD_3OD$ 49.0 ppm, $^{n}J_{C,H}$ 8 Hz).

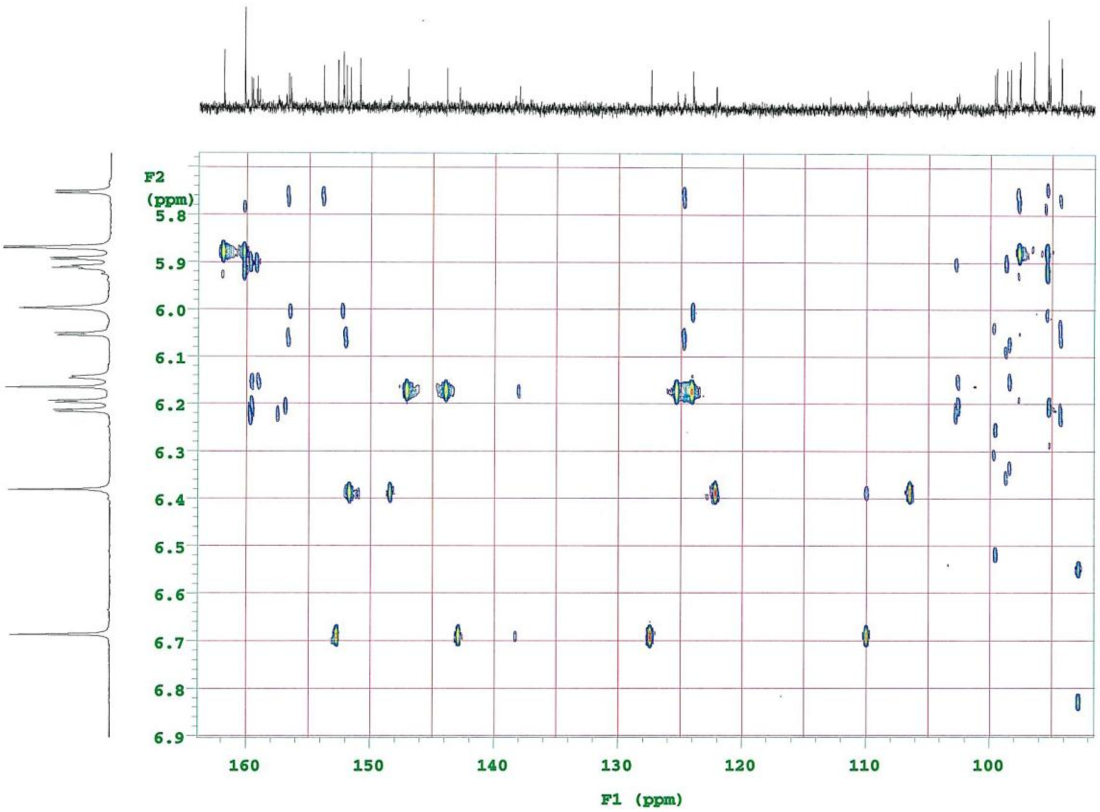

Figure S14. ^{1}H NMR spectrum of **2** in $(CD_3)_2SO$ (600 MHz, $(C\underline{H}D_2)_2SO$ 2.50 ppm).

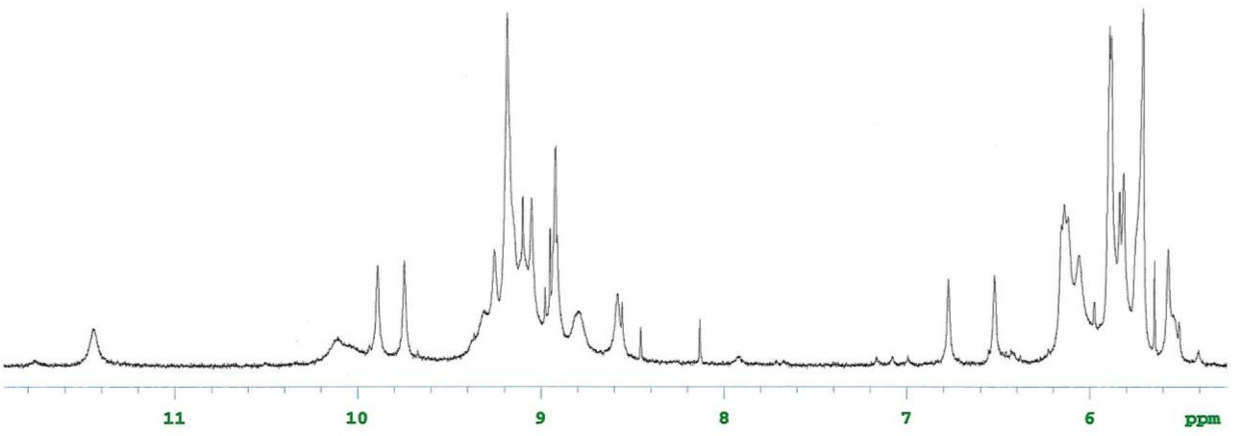

Supplement: Supplementary File 1 — Supplementary Information (PDF, 1119 KB) [file marinedrugs-11-00165-s001.pdf]
